# Endoscopic transorbital transpalpebral approach for a sphenoid wing meningioma

**DOI:** 10.3171/2025.1.FOCVID24179

**Published:** 2025-04-01

**Authors:** Marcello Magnani, Alessandro Carretta, Giacomo Sollini, Arianna Rustici, Sofia Asioli, Ernesto Pasquini, Diego Mazzatenta, Matteo Zoli

**Affiliations:** 1 Department of Biomedical and Neuromotor Sciences (DIBINEM), University of Bologna, Italy;; 2 Programma Neurochirurgia Ipofisi–Pituitary Unit, IRCCS Istituto delle Scienze Neurologiche di Bologna, Italy;; 3 ENT Unit, Azienda USL di Bologna, Ospedale Bellaria, Bologna, Italy; and; 4 Neuroradiology Unit, IRCCS Istituto delle Scienze Neurologiche di Bologna, Ospedale Maggiore, Bologna, Italy

**Keywords:** skull base, endoscopic transorbital approach, minimally invasive surgery, meningioma, transorbital neuroendoscopic approaches, TONES

## Abstract

Sphenoid wing meningiomas are slow-growing tumors arising in a region crossed by crucial neurovascular structures, for which surgery poses significant challenges. In the last decade, several cadaveric and clinical studies have proven the effectiveness of endoscopic transorbital surgery for skull base pathologies in accordance with the principles of minimal invasiveness. The ventral surgical corridor offered by this approach allows the early devascularization of the lesion without brain retraction, temporal muscle mobilization, and limited neurovascular structure manipulation. This 2D operative video describes the removal of a sphenoid wing meningioma using an endoscopic transorbital transpalpebral approach with minimal morbidity and quick recovery.

The video can be found here: https://stream.cadmore.media/r10.3171/2025.1.FOCVID24179

## Transcript

We present the case of an 80-year-old patient affected by a sphenoid wing meningioma treated with an endoscopic transorbital transpalpebral approach.

### 0:35 Clinical Presentation.

Patient past medical history was unremarkable. He underwent an appendicectomy in youth and an embolization of a vertebral tumor in 2017. In December 2023, he experienced a tonic-clonic seizure with persistent stereotyped right leg movements. The neurological examination did not reveal any alteration in terms of motor, sensory, or cranial nerve functions.

### 0:45 Neuroimaging Findings.

MRI demonstrated a contrast-enhancing lesion arising from the middle third of the greater sphenoid wing consistent with the hypothesis of meningioma.

### 1:20 Rationale for the Procedure.

In consideration of the clinical onset with a generalized seizure and the significant amount of perilesional edema, a first-line surgical strategy was more indicated compared to a wait-and-see or upfront radiosurgical options. Among the various surgical approaches amenable for the excision of the tumor, an endoscopic transorbital approach was adopted. The green lines highlight the surgical angles of attack, and the red dotted line identifies the posterolateral orbit wall to drill to access the tumor.

### 1:56 Risks and Potential Benefits for the Procedure.

The endoscopic transorbital approach presents several benefits. First of all, from a technical point of view, this approach offers a straightforward ventral surgical corridor to the paramedian and median skull base.^1–3^ This ventral corridor allows for an early devascularization of the tumor. It does not require brain retraction or temporal muscle mobilization and limits the manipulation of critical neurovascular structures. Then, since the surgical access is through a small incision on the superior eyelid crease, the following scar is less visible.^3^ Eventually, according to its minimal invasive nature, it is possible to achieve a quicker patient recovery and a reduced length of hospitalization.

The risks are represented by the reduced surgical maneuverability for the more deep and medial portions of the tumor and by the need of an extensive and specific training in endoscopic surgery.

### 2:55 Alternatives and Why They Were Not Chosen.

Other alternatives could be represented by proximally extended endoscopic transorbital approaches with hinge orbitotomy or complete orbitotomy or by the traditional microneurosurgical transcranial frontotemporal approaches.

In this case, given the location of the meningioma arising from the middle third of the greater sphenoid wing, an endoscopic transorbital approach with additional orbitotomy was not adopted since the benefits in terms of surgical maneuverability on the axial plane provided by the removal of the lateral orbit rim did not counterbalance the potential cosmetic and functional pitfalls related to a more complex reconstructive phase and to a longer surgical time.^4^ Conversely, in case of a more medial and deep-seated lesion, the increased surgical freedom provided by an endoscopic transorbital approach with additional lateral orbit rim osteotomy would have been crucial to perform a safe and radical resection.^5^ The traditional frontotemporal approach was not selected due to the potential technical, cosmetic, and functional disadvantages.

### 4:01 Description of the Setup.

The patient lies in a supine position, under general anesthesia, with orotracheal intubation. A 0° and 30° endoscope, an exoscope, neuronavigation, and intraoperative Doppler are adopted. Surgery is performed by an ENT surgeon with long experience in endoscopic surgery and oculoplasty and by a skull base neurosurgeon with long experience in endoscopic surgery.

### 4:30 Key Surgical Steps.

The endoscopic transorbital approach presents several key steps. First of all, the detachment of the periorbit from the lateral orbit wall, the identification of key anatomical landmarks such as the superior orbital fissure, the drilling of the lateral orbit wall, and the coagulation of the base of implant of the meningioma, which, in this case, it is the temporal dura itself. Then, the procedure advances with central debulking and detachment of the meningioma from the adjacent structures. At the end, the reconstructive phase is carried out with a duraplasty with dural substitutes and an abdominal fat graft.

### 5:13 Surgical Video.

The procedure begins with a skin incision at the level of the superior eyelid crease and further exposure of the superior orbital rim. Then, it proceeds with the detachment of the periorbita from the lateral orbit rim until the identification of the superior orbital fissure. The following step consists in the drilling of the posterolateral orbit wall to create the ventral corridor to the paramedian and median skull base. This core phase proceeds until the exposure of the temporal dura, which, in this case, represents the base of implant of the meningioma and, laterally, of the deep temporal fascia. The temporal dura is coagulated to obtain an early devascularization of the lesion. A dural incision in a circular fashion is performed, and further dissection of the tumor component jutting out toward the orbit is completed with scissors to obtain a piece of meningioma for the histological examination. Tumor removal phase begins with central debulking of the lesion aided by the use of ultrasonic aspirator to lighten the bulk of the tumor. Then, blunt dissectors are implemented to detach the lesion from the adjacent structures. The dissection proceeds with the alternation of ultrasonic aspirator and blunt instruments until the visualization of the interface between the lesion and the temporal pole arachnoid. Careful detachment of the capsule is performed even with the aid of wet cottonoids. Eventually, the last remnants of the meningioma are removed with the use of angled instruments and curettes. Tumor removal is then concluded with further coagulation of the base of implant and of the laterally extending dural tails. Hemostasis is made with dedicated bipolar cautery and performed on the congested temporal pole. Ultimately, hemostasis is achieved with the aid of hemostatic agents. Reconstruction consisted in the positioning of an overlay dural substitute with an abdominal fat graft to reduce the risk of CSF leak.

### 7:50 Disease Background.

The histopathological examination revealed a transitional grade 1 meningioma according to the 2021 WHO classification. Since the high Ki-67 proliferation index suggested an aggressive biological behavior, further FISH analysis and next-generation sequencing were conducted and eventually tested negative for pathogenic variants.

### 8:15 A Brief Overview of Clinical and Imaging Outcome.

Postoperative course was unremarkable. The patient referred a mild subjective diplopia without obvious extraocular muscle deficiencies and was discharged finally at home after 3 days. At 3 months’ follow-up, the patient presented in excellent condition. A resolution of the subjective diplopia was observed, and the gaze was preserved in all cardinal positions. The follow-up MRI confirmed the gross-total resection of the meningioma without further complications.

### 8:55 Conclusion.

Sphenoid wing meningiomas are slow-growing tumors arising in a complex anatomical region defined by the presence of several noble neurovascular structures, for which their surgical treatment poses significant challenges.^6^ Several cadaveric and clinical studies demonstrated the validity of endoscopic transorbital surgery for a minimal invasive treatment of skull base pathologies, with a growing body of indications.^3,4,7–9^ The ventral corridor of access to the median and paramedian skull base offered by the endoscopic transorbital approach represents a solid alternative compared to that of the traditional transcranial microneurosurgical approaches, with the advantages of no brain retraction, temporal muscle manipulation, and reduced cosmetic morbidity.^1–3^ The use of a standard superior eyelid endoscopic transorbital approach resulted in the gross-total removal of the tumor with minimal morbidity, reduced patient discomfort, and quick recovery. In selected cases and dedicated endoscopic skull base centers, this option can be considered as safe and effective, with the aim of achieving a gross-total resection of the tumor with regard to the principles of minimal invasiveness and morbidity.

## Disclosures

The authors report no conflict of interest concerning the materials or methods used in this study or the findings specified in this publication.

## Author Contributions

Primary surgeon: Pasquini, Zoli. Assistant surgeon: Carretta, Magnani, Sollini. Editing and drafting the video and abstract: Carretta, Magnani, Sollini, Rustici, Mazzatenta. Critically revising the work: Carretta, Rustici, Zoli. Reviewed submitted version of the work: Carretta, Magnani. Approved the final version of the work on behalf of all authors: Carretta. Supervision: Rustici, Pasquini. Pathologist: Asioli.

## Supplemental Information

### Patient Informed Consent

The necessary patient informed consent was obtained in this study.
